# Reduced Graphene Oxide and Its Modifications as Catalyst Supports and Catalyst Layer Modifiers for PEMFC

**DOI:** 10.3390/ma11081405

**Published:** 2018-08-10

**Authors:** Sergey A. Grigoriev, Vladimir N. Fateev, Artem S. Pushkarev, Irina V. Pushkareva, Natalia A. Ivanova, Valery N. Kalinichenko, Mikhail Yu. Presnyakov, Xing Wei

**Affiliations:** 1 National Research University “Moscow Power Engineering Institute”, 14, Krasnokazarmennaya st., Moscow 111250, Russia; pushkarev_as@outlook.com (A.S.P.); pushkareva_iv@outlook.com (I.V.P.); 2 National Research Centre “Kurchatov Institute”, 1, Akademika Kurchatova sq., Moscow 123182, Russia; fateev_vn@nrcki.ru (V.N.F.); ivanovana.1989@outlook.com (N.A.I.); kalinval47@mail.ru (V.N.K.); mpresniakov@gmail.com (M.Y.P.); 3 Semenov Institute of Chemical Physics of Russian Academy of Sciences, 4, Kosygina st., Moscow 119991, Russia; 4 Changchun Institute of Applied Chemistry CAS, Renmin str., 5625, Changchun 130002, China; gejj@ciac.ac.cn

**Keywords:** PEM fuel cell, electrocatalyst, reduced graphene oxide, RGO doping, platinum

## Abstract

Reduced graphene oxide (RGO) and RGO modified by ozone (RGO-O) and fluorine (RGO-F) were synthesized. Pt nanoparticles were deposited on these materials and also on Vulcan XC-72 using the polyol method. The structural and electrochemical properties of the obtained catalysts were investigated in a model glass three-electrode electrochemical cell and in a laboratory PEM fuel cell. Among the RGO-based catalysts, the highest electrochemically active surface area (EASA) was obtained for the oxidized RGO supported catalyst. The EASA of the fluorine-modified RGO-supported catalyst was half as big. In the PEM fuel cell the performance of RGO-based catalysts did not exceed the activity of Vulcan XC-72-based catalysts. However, the addition of an RGO-O-based catalyst to Vulcan XC-72-based catalyst (in contrast to the RGO-F-based catalyst) allowed us to increase the catalyst layer activity and PEM fuel cell performance. Possible reasons for such an effect are discussed.

## 1. Introduction

During the last two decades, interest in graphene-type materials (graphene, reduced graphene oxide (RGO), carbon nanotubes (CNT), nanofibers (CNF) and so on) as electrocatalyst supports for proton-exchange membrane (PEM) electrochemical systems [1] has been growing (see, for example, [2,3,4,5,6,7,8]). This interest is based on their higher electric conductivity in comparison with conventional carbon supports (i.e., Vulcan XC-72), which can facilitate electron transfer during electrochemical reactions and higher corrosion resistance. Moreover, large linear dimensions may permit them to form efficient microporous structures with improved reactants/product transport properties. In addition, the texture of graphene planes provides suitable sites for Pt impregnation that could potentially reduce the migration and agglomeration of Pt nanoparticles [4]. It is worth stressing that graphene-type materials (in particular RGO) seem more attractive in comparison with CNF and CNT as they have two surfaces available for catalyst deposition, while the internal surface of CNT and CNF is practically not accessible for catalytic particles. RGO-based materials may also increase the “traditional” electrode structure efficiency due to their higher electric and thermal conductivity, and the modification of textural properties catalyst layers [9,10,11,12,13,14]. Another factor is an increase of the Pt surface catalytic efficiency due to the interaction of Pt/Vulcan XC-72 particles with such additions in analogy with the mechanism of catalyst layer activation by additions of CNT activated by oxygen-containing (quinone–hydroquinone) groups [15,16,17]. Donor–acceptor properties of graphene-type carbon materials can play an important role. There is a large possibility of these properties’ modification by functionalization of the surface and doping with different elements (N, B, P, S, O, F) [18,19,20,21,22,23]. So, one has an ample opportunity to improve the catalysts’ and catalytic layers’ activity by graphene-type material modification.

In the present study we synthesized and studied the physical–chemical properties of RGO and RGO modified by ozone and fluoride, and Pt catalysts based on such supports. The effect of such materials’ addition to the catalyst layers based on Vulcan XC-72 on catalysts’ electrochemical parameters was also investigated.

## 2. Materials and Methods

### 2.1. RGO Synthesis and Modification

Graphite oxide was synthesized using a modified Hummers’ method [24]. The modification of the Hummers’ method and the RGO synthesis are described in detail in [4]. The method permits us to obtain RGO with an oxygen concentration up to 6 wt % after thermal reduction from graphene oxide. In the final stage, the heating of the dry graphite oxide strips was carried out at 700 °C in Ar with the addition of hydrogen (1–2 wt %) to decrease the oxygen concentration in RGO.

RGO doped with fluorine was obtained by graphite fluorination, carried out in a liquid iodine heptafluoride at 25 °C in an argon atmosphere. The graphite suspension in a fluorine precursor was maintained for 3 h and the excess of iodine heptafluoride (and iodine pentafluoride) was removed by freezing with liquid nitrogen. The obtained yellow product was heated at 500 °C in an Ar atmosphere for 1–2 h for the reduction and expansion of fluorinated graphite. The obtained fluffy black product was washed in deionized water and dried at room temperature.

RGO ozone treatment was carried out at 30 °C with an ozone–oxygen mixture produced by barrier discharge in ozone generator “MEDOZONE” UOTA-60-01. The ozone concentration was ca. 30–50 mg/L. The time of the treatment was ca. 40–50 min up to stopping the ozone absorption.

### 2.2. Pt-Based Catalyst Synthesis

The preparation of the platinum black catalyst on a carbon support was carried out using the ethylene glycol (EG) reduction method and H_2_PtCl_6_ as a Pt precursor. The synthesis was carried out in a Drexel flask placed in an ultrasonic bath with argon flowing in the flask. A suspension consisting of a support (440 mg), H_2_PtCl_6_ (156 mg of Pt), and a mixture of ethylene glycol-deionized water (1.2:1) (110 mL), isopropanol (1.5 mL), and sodium dodecyl sulfate (30 mg) was sonicated at 22–25 kHz for 5 min. NaHCO_3_ (300 mg) was used to raise the pH value to 9. The resulting suspension was poured into a Drexel flask at 70–80 °C and then sonicated at 37 kHz. The synthesis was carried out for two hours with argon flowing at a constant temperature. During this time reduction and soot precipitation of Pt took place. The reduction took place in two steps:

Pt (4) → Pt (2) (1)

Pt (2) → Pt (0) (2)

The resulting catalyst was carefully washed with bi-distilled water and dried in the oven at 70 °C overnight.

### 2.3. RGO and Pt/RGO Characterization

The specific surface was detected by low-temperature nitrogen adsorption using TRISTAR (Micromeritics, Norcross, GA, USA).

The morphology of the catalysts was investigated using emission scanning electron microscopy (SEM, Helios NanoLab 600i, FEI, Hillsboro, OR, USA) equipped with an energy-dispersive X-ray (EDX) spectrometer (EDAX, Mahwah, NJ, USA). All SEM images were obtained in secondary electrons (SE) detection mode.

After that the specimens were studied in a scanning/transmission electron microscope (S/TEM) Titan 80–300 (Thermo-Fisher Scientific, Beverly, MA, USA) equipped with a spherical aberration (Cs) electron probe corrector, and high-angle annular dark field (HAADF) (Fischione, Export, PA, USA) detector, EDX spectrometer (EDAX, Mahwah, NJ, USA), and post-column Gatan Image Filter (GIF; Gatan, Pleasanton, CA, USA). The TEM analyses were performed at 300 kV. This study provides information on Pt nanoparticles and RGO with atomic resolution.

Samples for TEM analysis were prepared by sonicating a certain amount of catalyst in ethanol for 10 min and precipitating the solution on a standard Cu grid covered with Lacey© carbon film. This technique resulted in the detection of some Cu impurities in the catalysts. 

IR spectra were recorded with a Spectrum Two (Perkin-Elmer, Waltham, MA, USA) spectrometer in a “pass-through” mode using a Microfocus accessory (Perkin-Elmer) and a substrate from ZnSe with a diameter of 13 mm and a thickness of 2 mm.

### 2.4. Catalysts Electrochemical Characterization

The cyclic voltammograms (CVs) were measured in Ar-saturated 1M H_2_SO_4_ at 25 °C using a conventional three-electrode glass cell. The measurements were performed using a Solartron 1285 (Solartron Analytical) potentiostat. A saturated Ag/AgCl/KCl (SSCE) and a Pt wire were used as the reference and counter electrodes, respectively. A glassy carbon disk electrode with an area of 0.4 cm^2^, with a catalytic layer deposited on it from an ethanol solution of ionomer (Nafion^®^), served as a working electrode. The potential range −0.17 to 1.2 V vs. SSCE at a sweep rate of 20 mV/s was applied. The catalyst’s electrochemically active surface area (EASA) was determined using the region of hydrogen adsorption–desorption peaks as described in [25,26,27].

### 2.5. Membrane Electrode Assemblies (MEAs) Preparation and Testing

MEAs with a geometrical area of 7 cm^2^ were fabricated using Sigracet^®^ GDL 38 BC (SGL Carbon GmbH, Wiesbaden, Germany) carbon-fiber paper disks (ca. 0.3 mm thick, 30 mm in diameter and ca. 80% effective porosity) as cathode and anode gas diffusion electrodes (GDL) (see also [5]).

Pt supported on Vulcan XC-72 or RGO (RGO-O and RGO-F as well) or supported on the Vulcan XC-72 mixture with RGO (RGO-O and RGO-F as well) with 40 wt % of Pt were tested. Pt supported on Vulcan XC-72 was used as the anode catalyst in all cases. Cathode and anode Pt loading was 0.6 and 0.2 mg/cm^2^, respectively. The dry weight ionomer content was 15 wt %.

The anodic and cathodic catalytic inks with a certain amount of catalyst and Nafion^®^ solution in ethanol were sonicated and air-sprayed over the surface of GDL at 90 °C. 

PEMFC testing was carried out in a laboratory cell as described earlier [5,11].

Electrolytic-grade hydrogen and oxygen were used as the fuel and oxidant for the fuel cell, respectively. The MEAs were made using the following procedure: after assembling to the hardware the MEA was kept at 90 °C for 10 min to facilitate the bonding of electrodes with the membrane. Nafion^®^ 115 was used in all MEAs. The cell was supplied with H_2_ and O_2_ gases at 100% humidity at the fuel cell operating temperature. The cell was operated at 0.5 V until the current reached a steady-state value (±3%) before polarization curve recording. Polarization curves were recorded at 1 bar gauge gas pressures and a cell temperature of 60 °С. The O_2_ flow was 10 mL/s to remove the excess of water.

## 3. Results and Discussion

### 3.1. Structural Studies of Pt Electrocatalysts Based on RGO

**Figure 1:**
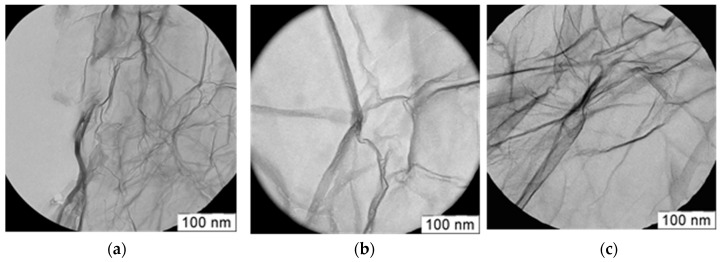
TEM photo of RGO after ozone treatment (**a**), untreated RGO (**b**), and RGO after fluorination (**c**).

The RGO treatment by ozone and iodine heptafluoride did not influence the material structure too significantly. The surface structure of RGO and RGO after ozone (RGO-O) and fluorine (RGO-F) treatment is shown in Figure 1.

One can see that the surface structure is rather similar and ozone treatment did not destroy the RGO structure. Some folds of the RGO surface are typical for these materials. A similar structure was observed and after fluorine modification.

After ozone and fluorine modification, we observed a decrease in the RGO specific surface (from 570 to 520–540 m^2^/g in the case of oxygen and fluorine treatment, respectively) so the destruction of RGO during the treatment was not large.

The RGO composition before and after treatment is shown in Table 1.

The data in Table 1 show that the chemical modification (formation of surface compounds and doping) of RGO takes place during the treatment. However, RGO retains its structure during fluorination and oxidation. A certain decrease in the specific surface area can be attributed to the oxidation of the most active/defective surface areas that react chemically with the formation of gaseous components, for example, carbon dioxide, in the case of ozone.

According to the IR-spectroscopy after the RGO ozone treatment, the number of carbonyl groups increased, which is reflected in the increase of adsorption band intensity in the 1650–1800 cm^−1^ and 1400–1650 cm^−1^ regions. Also, ozone treatment leads to the adsorption band intensity increasing at 1618 cm^−1^, which can be attributed to C=O vibrations of the appeared β-diketones. However, the exact surface group composition was difficult to determine.

**Figure 2:**
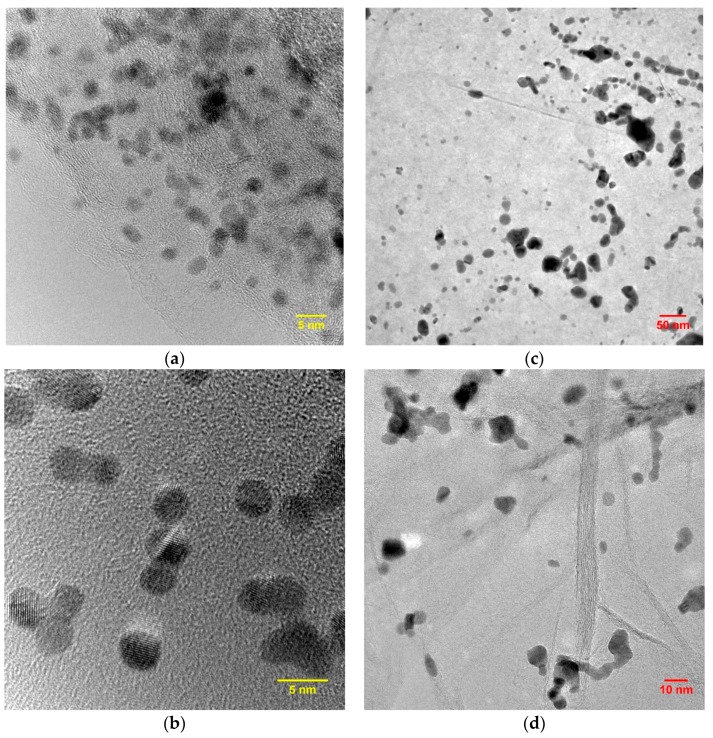
TEM-micrographs of Pt catalysts deposited on RGO modified by ozone (**a**,**b**) and fluorine (**c**,**d**).

Synthesis of the platinum catalyst on all types of materials was carried out in the same manner each time. For comparative studies, catalysts containing 40 wt % (Pt40/C) were chosen. The structure of Pt catalysts synthesized by polyol method on RGO is shown in Figure 2. As seen from Figure 2, the particles have the same shape and are evenly distributed over the RGO surface. In all the cases the Pt particles had a rather uniform distribution on the RGO surface and a rather narrow size distribution (Figure 2).

**Figure 3:**
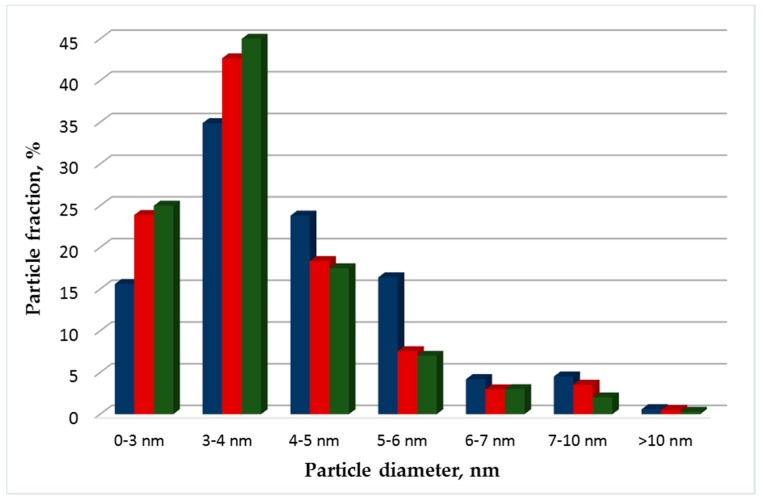
Size distribution of the obtained platinum particles for Pt40/RGO-O (**green**), Pt40/RGO-F (**blue**), and Pt40/RGO (**red**).

The obtained Pt particles supported on Vulcan XC-72, RGO and RGO-O have an average size of 2–6 nm, a spherical shape, and are evenly distributed on the support surface. However, the average particle diameter of Pt/RGO-F (Figure 3), calculated using the ImageJ software [28], was 5.9 nm. For Pt/Vulcan XC-72, Pt/RGO, and Pt/RGO-O, the average particle size was quite similar and much smaller (3.6–3.8 nm) than for Pt/RGO-F particles. The particle size distribution for Pt/Vulcan XC-72, Pt/RGO, and Pt/RGO-O supports was quite similar. The Pt particle size has a significant influence on the kinetics and durability of the fuel cell cathode catalyst [29]. Maximum catalytic activity was found at a Pt particle size of around 2 nm, while the highest specific activity was obtained at a Pt particle size of about 3 nm [30]. On the other hand, to get better catalyst durability Pt particles size should be larger than 3.5 nm [31]. An analysis of the above experimental results shows that most (more than 80%) of the particles have a diameter less than 5 nm, which is close to the optimum value (4–5 nm) [29].

### 3.2. Electrochemical Studies of Pt Electrocatalysts Based on RGO

**Figure 4:**
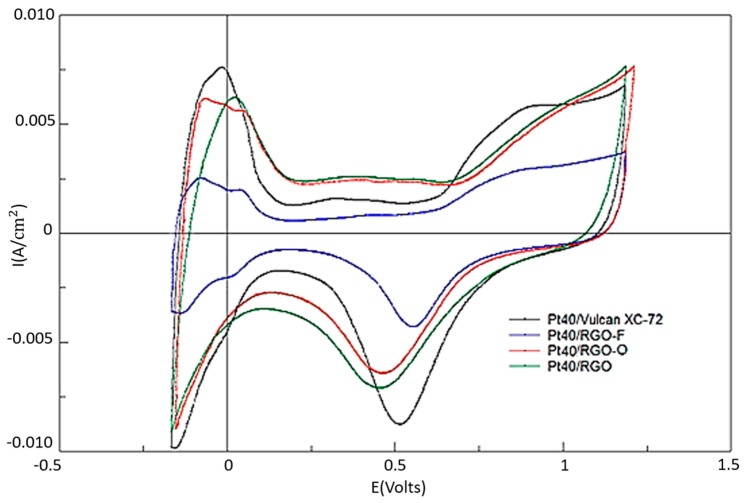
Cyclic voltammograms of electrodes based on Pt40/Vulcan XC-72, Pt40/RGO-O, Pt40/RGO-F and Pt40/RGO (1M H_2_SO_4_, 25 °C, 20 mV/s).

Figure 4 presents the results of cyclic voltammetry studies of the Pt40/Vulcan XC-72, Pt40/RGO, Pt40/RGO-O, and Pt40/RGO-F catalysts. The calculated values of the electrochemically active surface area (EASA) of the synthesized electrocatalysts were 57, 48, 51, and 20 m^2^/g Pt, respectively. These EASA values are a bit smaller than expected, taking into account the Pt particles’ size. This could be attributed to a poor utilization of the catalyst surface. When graphene-like materials are used as catalyst supports, the method of catalyst synthesis and the type of graphene precursor significantly influences the EASA of the resulted catalysts [32,33,34,35]. For instance, the catalyst utilization can change from 28% to 93% depending on the synthesis procedure for graphene-supported catalysts [32].

The formation of functional oxygen-containing groups on the RGO surface should promote the reagents’ sorption and more effective nucleation during catalyst synthesis, i.e., should reduce the size of the catalyst particles. However, the EASA for RGO-O is quite similar to that of an RGO-based catalyst. One can suppose that during the surface oxidation by ozone, with the formation of the functional oxygen-containing groups, the most chemically active surface sites of RGO, which also act as nucleation centers for the platinum catalyst, are oxidized. In such a case, the total number of nucleation centers is not increased too much. For RGO-F-supported electrocatalysts, EASA is about half that of the EASA of catalysts supported on other carbon materials. This could be explained by the high acceptor properties of fluorine atoms and the reduced surface availability for the reagents sorption during the catalyst synthesis. This leads to an increase in the Pt particle size and to a decrease in the catalyst EASA.

However, a decrease in Pt atoms’ ability to absorb hydrogen due to fluorine doping can also take place. In general, the main regions (peaks) of the obtained CVs are in good agreement with the literature data for Pt carbon-based catalysts [25,26,27]. However, for an RGO-supported catalyst the hydrogen adsorption and desorption peaks for the various crystalline Pt faces (i.e., Pt (111), Pt (200)) are not particular clear in the potential range from −0.17 to −0.1 V vs. SSCE. The possible reason may be the specific interaction of platinum particles with the unmodified RGO surface or impurities on the surface.

### 3.3. Fuel Cell Testing of RGO-Supported Pt Electrocatalysts

**Figure 5:**
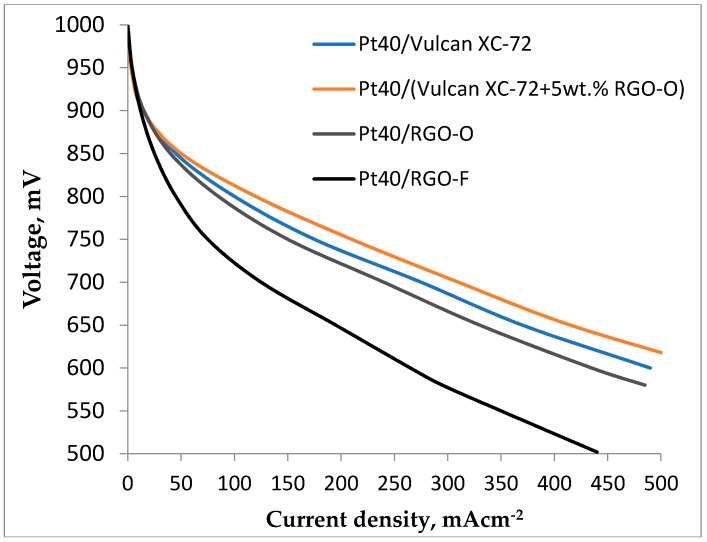
PEMFC polarization curves at 60 °C for MEAs with different cathodes: Pt40/Vulcan XC-72 (**blue**), Pt40/RGO-O (**green**), Pt40/RGO-F (**black**) and Pt40/Vulcan XC-72 with 5 wt % RGO-O addition.

The study of the above-described electrocatalysts as a cathode material in proton exchange membrane fuel cells (PEMFC) demonstrated that the activity of RGO and RGO-O-based electrocatalysts is quite close to that of Vulcan XC-72-based ones (Figure 5). RGO and RGO-O-based catalysts have almost the same activity in the oxygen reduction reaction (ORR) in PEMFC. The current density in the case of RGO-O was only 1–2% higher than in the case of RGO. The activity of the RGO-F-supported catalyst is much lower due to the low EASA.

The EASA certainly plays an important role, but the accessibility of the catalyst particles and the catalyst utilization can be lower in the case of RGO-based materials due to their layer-like structure [4,8]. The reduced graphene sheets tend to form irreversible agglomerates because of Van der Waals interaction and even form graphite during the reduction of RGO suspension or during the drying process [10]. However, the metal nanoparticles deposited on the RGO layer surface may prevent these processes by the formation of graphene particle composites [36]. Another possible explanation is a horizontal stacking of graphene layers, even with a Pt catalyst, during the electrode fabrication (spraying or blading of catalyst ink) and cell assembly [8], which is not easily detectable during experiments in a liquid electrolyte. This could be one reason for the lower activity of RGO-based catalysts.

When using RGO- and RGO-O-supported catalysts as additives for Vulcan XC-72-supported catalysts, some increase in the catalytic layer activity (a little bit larger in the case of RGO-O) was observed. This could be attributed to the decrease of the catalytic layer ohmic resistance, more effective contact of RGO particles with Vulcan XC-72, and the inclusion of a greater number of catalytic particles in the electrochemical process at operating current density [5,9,13,14]. For example, in [11] the authors postulated that small RGO addition to the catalyst layer changes the catalyst layer structure, i.e., increases the porosity of the active layer and its permeability to gases, and decreases the percolation limit for ionic conductivity. Moreover, in [37] graphene addition into the catalyst layer effectively improved the durability when operating at a high current density. By impedance measurements the existence of a conductive graphene network was demonstrated.

Our electron conductivity measurements of the catalyst layers by the four-probe method [38] showed that the RGO-supported and Vulcan XC-72 with RGO and RGO-O additive-supported catalyst layer resistivity is a bit (less than 2–4%) smaller than the resistivity of the Pt/Vulcan XC-72 catalyst layer. Therefore, decreasing the catalyst layer resistivity can play a role in the increase of Vulcan XC-72 with RGO and RGO-O additives’ catalyst layer activity.

The effective participation of oxygen-containing groups in the electron transfer on a platinum catalyst, proposed in [16,17,18], is also possible. This effect of the catalyst layer activity increase is absent in the case of an RGO-F-supported catalyst, which has the lowest activity. It could be associated with its lowest EASA value. On the other hand, it is obvious that the strong acceptor fluorine properties could result in a negative effect on the participation of RGO particles in the electron transfer in the catalyst layer and the electrocatalytic process. RGO-F-based catalyst additions to the catalyst layer even had a small negative influence.

## 4. Conclusions

This paper presents a study of the physicochemical and electrocatalytic properties of RGO-, RGO-F-, RGO-O-, and Pt-based catalysts. For the selected modification conditions at which the concentration of oxygen and fluorine atoms in the RGO reached 8.37 and 6.6 wt %, respectively, the RGO particle keeps its layered structure. The RGO specific surface area decreased insignificantly after the modification. On the one hand, oxygen modification of the RGO surface leads to a slight decrease in the average Pt particle size. However, in the case of fluorine modification of RGO, the average particle diameter increases. This may be due to the strong acceptor fluorine properties of the RGO-F support, which lead to a decreasing ability to absorb the Pt precursor during the catalyst synthesis process.

All synthesized RGO-based catalysts demonstrated lower performance in comparison with the Vulcan XC-72-supported one. However, RGO- and RGO-O-based catalyst addition to the Vulcan XC-72-based electrocatalyst led to an increase in the electrochemical activity of the catalytic layer, which is slightly higher in the case of RGO-O. The observed catalytic activity increase could be attributed to the increase in electric contact efficiency between catalyst particles. The effect of the Pt catalyst on Vulcan activation due to RGO-O oxygen groups’ participation in the electron transfer may also play a role.

So, the modification of RGO (and probably other graphene-type materials) by elements with different donor–acceptor properties can significantly change its electrochemical properties and has to be examined more carefully.

## Figures and Tables

**Table 1:** RGO compositions.

Material	RGO	RGO-F	RGO-O
Element	wt %		
C	97.15	91.45	91.63
O	2.85	1.95	8.37
F	0.00	6.60	0.00
